# [Corrigendum] Morroniside protects SK-N-SH human neuroblastoma cells against H_2_O_2_-induced damage

**DOI:** 10.3892/ijmm.2025.5501

**Published:** 2025-02-10

**Authors:** Jing-Xing Zhang, Rui Wang, Jin Xi, Lin Shen, An-You Zhu, Qi Qi, Qi-Yi Wang, Lun-Jun Zhang, Feng-Chao Wang, He-Zuo Lü, Jian-Guo Hu

Int J Mol Med 39: 603-612, 2017; DOI: 10.3892/ijmm.2017.2882

Following the publication of the above article, an interested reader drew to the authors' attention that, in Fig. 2D on p. 606, which showed the results of cellular morphological experiments, two pairs of data panels were overlapping, such that data which were intended to show the results obtained under different experimental conditions may have been derived from the same original sources.

The authors examined their original data, and realized that this figure had been assembled incorrectly (they were also able to send the data underlying this figure on to the Editorial Office for our inspection). The revised version of Fig. 2, now showing alternative data from one set of the repeated experiments, is shown on the next page. The authors confirm that the errors associated with this figure did not have any significant impact on either the results or the conclusions reported in this study, and all the authors agree with the publication of this Corrigendum. The authors are grateful to the Editor of *International Journal of Molecular Medicine* for granting them the opportunity to publish this Corrigendum; furthermore, they apologize to the readership of the Journal for any inconvenience caused.

## Figures and Tables

**Figure 2:**
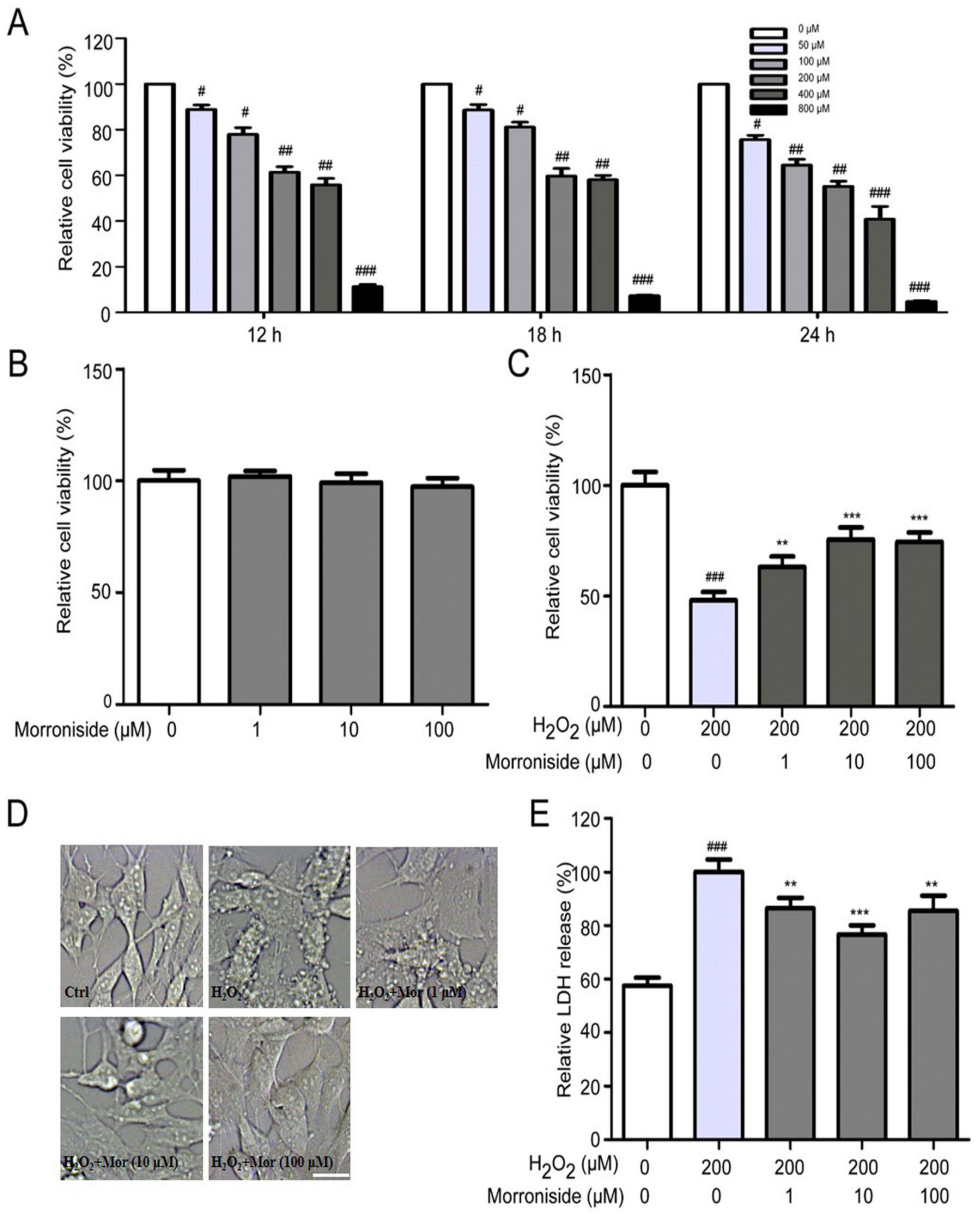
(A) Effects of H_2_O_2_ on SK-N-SH cell viability. Cell viability was reduced in a dose- and time-dependent manner by treatment with H_2_O_2_ (200 µM for 24 h). (B) Effects of morroniside on SK-N-SH cell viability. Morroniside treatment did not affect cell viability at concentrations of 1, 10 and 100 µM. (C) Effects of morroniside on H_2_O_2_-induced SK-N-SH cell death. Pretreatment with morroniside (1–100 µM) for 24 h restored viability in cells exposed to H_2_O_2_(200 µM) in a dose-dependent manner. (D) Morroniside inhibits morphological changes resulting from H_2_O_2_ treatment. Scale bar, 20 µM. (E) Morroniside suppresses H_2_O_2_-induced lactate dehydrogenase (LDH) release in SK-N-SH cells. Cells were pretreated with indicated concentrations of morroniside (1–100 µM) for 24 h following treatment with 200 µM H_2_O_2_ or no treatment for 24 h. LDH activity in the medium was measured. Data represent mean ± SD (n=3). ^#^P<0.05, ^##^P<0.01 and ^###^P<0.001 vs. control group; ^**^P<0.01 and ^***^P<0.001 vs. H_2_O_2_-treated group.

